# Good Trouble Along the Winding Road: Disruption and Accomplishment

**DOI:** 10.1093/geroni/igab046.159

**Published:** 2021-12-17

**Authors:** Harvey Sterns, Joseph Ruby

**Affiliations:** 1 The University of Akron, Akron, Ohio, United States; 2 Joe Ruby Consulting, Akron, Ohio, United States

## Abstract

The establishment of a accreditation body for gerontology degree programs was seen as disruptive. Many key leaders were against creating such a body and wanted this to be delayed or to never happen. In 2012, the AGHE Accreditation Task Force was established with a Competency Work Group and an Organization Work Group..There have been 5 programs evaluated with a number of schools/university currently in process. The task force filed documents for creating a legal entity and obtaining non-profit status for the new Accreditation for Gerontology Education Council. The Task Force developed the dimensions for program evaluation based on the Competencies and shared information with AGHE members. The Task Force obtained start-up funding for the organization and identified the first programs for accreditation and has been providing outreach and guidance to new programs. There is continual refinement of the process.

